# Virtually There: A Framework for Immersive Technology to Enable Global Health Engagements–Pioneering Frontiers in Medical Readiness and Global Health Diplomacy

**DOI:** 10.1089/jmedxr.2024.0030

**Published:** 2025-03-21

**Authors:** Janette Arencibia, Bianca B. Miranda, Jenna Watts

**Affiliations:** ^1^ Sea Suite Solutions, LLC, St. Petersburg, Florida, USA.; ^2^ HEALTHTECH BR, Sao Paulo - Brazil.; ^3^ Ringling College of Art and Design, Sarasota, Florida, USA.

**Keywords:** virtual, augmented, extended, immersive technology, Global Health Engagement

## Abstract

This article examines how virtual reality, augmented reality, and mixed reality are transforming health care education and training, especially in Global Health Engagements (GHEs). It highlights how these technologies can overcome geographic barriers, expand access to medical education, and foster international cooperation. The article introduces a framework, based on the U.S. Department of Defense’s “Doctrine, Organization, Training, Material, Leadership, Personnel, and Facilities” model, to guide the adoption of these technologies and align them with U.S. GHE goals. The framework covers key areas such as technology terminology, equipment setup, secure online participation, user experience, system capabilities, and cultural considerations. Its goal is to encourage innovation and address challenges to enhance global health outcomes through immersive technologies.

## Introduction

The fast-paced development of technology has created new opportunities to improve health care education and training. Traditional methods are often restricted by distance, high costs, and logistical difficulties. In contrast, XR technologies provide innovative solutions such as remote education, affordable simulations, advanced surgical training, and personalized learning experiences. This article addresses an innovative approach to overcome these obstacles by leveraging immersive technologies—virtual reality (VR), augmented reality (AR), and mixed reality (MR)—collectively known as extended reality (XR).

The primary goal of this article is to propose a comprehensive framework for integrating XR technologies into health care education and training for international populations. This framework supports Global Health Engagements (GHEs) between the United States and partner nations (PNs) by offering a standardized approach to adopting immersive technologies. By properly implementing medical extended reality (MXR) technology as a training tool, widespread adoption is encouraged, empowering GHE initiatives by connecting medical professionals and learners globally. Traditionally, GHEs involve travel, supplies, and other resources. However, MXR can create virtual learning environments that allow immersive interactions without the need for travel. Additionally, these virtual platforms provide virtual supplies, eliminating the need for resupply and reducing medical waste. An example of this is subject-matter expert exchanges between medical providers, where MXR enables real-time collaboration and learning across borders.

When GHE initiatives become clearly prioritized during crises such as COVID-19 and other global health threats, it is essential to proactively prepare and develop infrastructure designed to handle situations that require collaboration. GHE partnerships facilitate the sharing of vital information, resources, and best practices, enabling a coordinated international response. By combining efforts, countries can improve surveillance systems, accelerate the development and distribution of diagnostics, vaccines, therapeutics, and strengthen health care infrastructures. In yet another example, efforts to collaborate between Colombia and the United States for best practices in critical care had global health professionals and physicians seeking virtual solutions to enable real-time, personable teaching and training opportunities. These circumstances encouraged the authors to develop a framework for consideration in implementing immersive tech to achieve global health goals.

Including immersive technologies is important because it has the potential to transform health care training by creating realistic, virtual, high-fidelity training experiences. Virtual scenarios serve as an effective tool to enhance the preparedness of health care professionals for emergency and high-pressure situations.^1^ The presented framework focuses on key areas, including understanding immersive technology terminology, onboarding equipment, and ensuring secure online participation. By addressing these factors, the framework aims to maximize the effectiveness of XR technologies, enhancing global health readiness and outcomes. In summary, this article provides a blueprint for integrating innovative methods into health care education and training to support global health activities more effectively.

## Background

The rise of immersive technology has led to the creation of professional associations that encourage global collaboration among health care professionals. Groups such as the International Healthcare Virtual Reality Association (IHVRA) and the American Medical Extended Reality Association (AMXRA) help promote the use and development of immersive technology in health care. These organizations are vital for advancing the capabilities and trends of this technology. To maintain awareness and collaboration among international partners, developers, health care professionals, and educators, the authors—active participants in these organizations—propose a framework for integrating immersive technology into health care training. This framework aims to ensure successful adoption by clarifying the types of immersive technologies and matching them to users’ needs, avoiding failures caused by mismatched tools and requirements.

## Discussion

While immersive technology is gaining traction in medical training, its widespread implementation is still in the early stages. For instance, early efforts to conduct medical subject-matter expert exchanges between the United States and Latin American counterparts faced challenges, particularly with equipment compatibility. Without a clear framework in place, key requirements such as connectivity were only addressed after problems arose.

By developing a structured framework, both the United States and PNs can overcome these technical challenges and improve clinical aptitude. A well-designed system would ensure compatibility, streamline connectivity, and provide guidance on equipment setup and usage. This would result in smoother exchanges of expertise, enabling health care professionals to enhance their skills, share knowledge, and collaborate more effectively across borders. Ultimately, this approach has the potential to significantly raise the level of clinical expertise in both the United States and PNs, particularly in high-pressure and emergency situations.

Within GHE initiatives, three-dimensional (3D) virtual environments offer dynamic platforms for scientific exchange. They enable health care professionals, researchers, and students to interact with 3D data visualizations, engage in real-time collaborative efforts within shared digital spaces, and perform hands-on practice of complex procedures. These immersive platforms facilitate deeper discussions, enhance skill acquisition, and promote innovative practices^2^; offering a more personalized and engaging experience often missing in traditional 2D screen online interactions. The possibility of integrating artificial intelligence (AI) driven real-time ambient dictation and language translation into these platforms can also effectively eliminate language barriers, enabling smooth communication among diverse international teams.^3^ Expanding even further the possibilities of collaboration among international peers.

The evolving landscape of technology and immersive experiences have become a cornerstone for innovation, offering various types of applications that extend beyond the traditional use of these technologies as entertainment. In attempting to introduce immersive technologies to achieve global health initiatives, it became clear that a guide for implementation is necessary. For those unfamiliar with immersive capabilities, the benefits may seem complex without a clear framework to reference. The term “extended reality” (XR) has emerged as a comprehensive designation for these technologies, encapsulating VR, AR, and MR, each representing a unique convergence of the digital and physical worlds.

VR, perceived as the most immersive form of XR, transports users into entirely artificial environments, rendering the real world obsolete for the duration of the experience. Through head-mounted displays and the use of handsets users can navigate and interact within created environments, achieved through the illusion of depth and space, creating a sense of presence that can evoke intense emotional and physical responses akin to those triggered by real-world experiences.^4^

In contrast, AR augments the reality perceived by overlaying digital information upon it, visible through a spectrum of devices. It doesn’t sever ties with the physical world, but rather enhances it, allowing us to process our environment enriched with digital augmentation. This has seen widespread adoption in consumer applications, as well as in various industries, where enhanced interaction with the real world can significantly improve efficiency and outcomes.

MR, the combination of AR and VR, blends digital content with the real world in such a seamless manner that the two can interact and coexist within a single user experience. This integration is made possible by sophisticated technologies that enable virtual objects to be obscured by real-world experiences, maintaining a coherent spatial relationship that is critical for the authenticity of the interaction. GHE activities that begin with either AR or VR might consider a transition to MR scenarios to enhance user experiences beyond the scope of either AR or VR. The authors acknowledge the challenge of communicating the differences between these technologies without having first experienced either VR or AR, yet another reason for publishing a suggested framework. For a better understanding of the reality-virtuality continuum, a review of the taxonomy work done by Milgram and Kishino in 1994 provides an orientation to the concepts of MR.^5^

These features enrich the user experience and expand the potential for immersive experiences in practical applications that, in some instances, are already in use to familiarize health care professionals with staff orientation in trauma environments.^6,7^ Many trauma centers have implemented AR to provide tailored simulation training for health care professionals, including in-unit CPR and crash cart orientation.^8,9^ Simulation centers allow educators to develop customized training sessions for nursing staff, enabling them to familiarize themselves with emergency equipment, such as crash carts, in a controlled, realistic digital setting. The use of AR tools simulates a real-world scenario without disrupting live medical environments.^10^ This initiative abbreviates training time, improves proficiency, and improves emergency preparedness and patient safety through innovative learning platforms.^11,12^

The integration of immersive technology to facilitate medical training programs requires special consideration and attention. As technology evolves, designers aim to simplify this process by creating intuitive user interfaces that allow users to understand and use the technology independently. Therefore, to ensure an effective implementation of immersive technology in medical training programs, a framework is necessary. The framework presented in Table 1 incorporates doctrine/policy, organization, training, material, leadership and education, facilities, and personnel.

**Table 1.** XR Implementation and Training Framework

Capability	None	Minimal	Moderate	Significant	End state
	0	1	2	3	4
Doctrine/policy	□ No familiarity with XR technology □ No support for utilization □ XR undefined, no policy	□ Some familiarity with XR technology □ Previous instruction on XR training □ Suggested/approved XR strategy/implementation □ Introduce initial policy drafts that recognize XR as a potential training tool	□ Request for XR program and training support □ XR is adopted as a teaching modality □ Implement XR-specific guidelines for pilot programs	□ XR program is supported by the governing entity □ Program has training/scenario manuals □ XR comprehension standards are outlined □ Implement XR-specific guidelines for pilot programs	□ Provides guidance on XR doctrine and policy □ XR mandated; requires sustained program □ Requires XR equipment and upgrades every 2–3 years □ Formalize XR policy, integrating it into doctrine for training and operational use
Organization	□ No centralized structure or organization to coordinate/implement XR	□ Identify a point of contact or champion for XR within the organization	□ Establish formal organizational roles and teams dedicated to XR	□ Participate in international XR forums and collaborations	□ Official entity demonstrates and participates in professional forums □ Fully integrated organizational participation in global XR initiatives
Training	□ No XR training program □ No identified training objectives	□ Basic familiarization training for a select group of personnel	□ Develop structured XR training programs for specialized roles	□ Implement comprehensive training across the organization with regular updates	□ Establish sustained, organization-wide training that is continuously updated and aligned with operational needs
Material	□ No XR equipment □ No storage capability	□ Acquire initial XR equipment for pilot testing	□ Expand across to XR systems and develop a support and maintenance structure	□ Ensure organization-wide access to XR technology, with robust support and logistics □ XR programming and support funding	□ Full access to advanced XR equipment, with continuous upgrades and support
Leadership and education	□ No XR-trained staff □ No professional association/membership affiliation	□ Expose leadership to the potential of XR through briefings and workshops	□ Develop leadership-focused training on XR management and oversight □ Organizational emphasis on XR-trained clinicians	□ Ensure leadership is proficient in managing XR initiatives and is leading the organizational shift □ Mandated and supported XR training at all leader schools and introductory/fundamental training □ Use case identification	□ Full leadership engagement in ongoing XR development, supporting innovation and strategic use □ Program has a designated career track that includes XR □ Organization sends medical personnel to professional XR development courses
Personnel	□ No personnel identified for training □ No interested personnel	□ Identify key individuals for initial XR training □ Small percentage trained (<2%) in XR familiarization □ Part-time trainers/additional duty trainers	□ Implement formal training program for broader range of personnel □ 2–10% trained and a training team established	□ Require mandatory certifications for personnel involved in XR initiatives □ Full-time XR trainers on staff	□ Establish fully trained workforce with mandatory certifications and ongoing professional development in XR
Facilities	□ No designated area for XR training □ No designated area for equipment □ No dedicated internet	□ Create designated pilot spaces for XR □ Power and internet availability □ Secure storage for materials	□ Expand facility support for XR in key operational areas □ Designated classroom □ Random scheduled courses	□ Develop dedicated XR facilities at regional levels □ Regularly scheduled courses with incorporated XR learning opportunities	□ Establish fully operational regional centers for XR training and operations within health care facilities □ XR capability is located regionally □ Facility has implemented XR training

XR, extended reality.

### Framework for implementing MXR

To ensure the effective implementation of immersive technology in medical training programs, a comprehensive framework is necessary.

### Doctrine and policy

Doctrine and policy play a critical role in program implementation, particularly within structured organizations, as they provide a clear vision and goals for the use of immersive technology. They ensure that all stakeholders have a common understanding of the purpose, objectives, and expected outcomes of the program. Policies ensure that immersive technology is integrated safely and effectively into medical training programs.

Policies should address several key elements:

- Equipment use and inventory
- Privacy and security
- Data management
- Intellectual property rights

During the development phase, it is important to involve cybersecurity, legal, and compliance teams. Clear guidance should also be provided regarding the use of immersive technology for accreditation and certification purposes. By aligning policies with doctrine, organizations can create a cohesive framework that supports the successful integration of immersive technologies into their training and operational practices.

**Key takeaway:** Aligning policies with doctrine creates a cohesive framework that supports the successful integration of immersive technologies into training and operational practices.

### Organizational structure

The organizational structure of an immersive technology program is crucial as it delineates roles and responsibilities, ensuring that individuals leading the program are aligned with the overarching goals. Effective coordination among staff is essential for fostering innovation and implementing improvements within the program.

To enhance their capabilities, individuals responsible for program implementation should engage with professional organizations such as the International Virtual Reality Healthcare Association and the AMXRA. These memberships offer access to best practices and valuable networking opportunities with peers in the field, facilitating the successful development and execution of immersive technology initiatives.

**Key takeaway:** A well-defined organizational structure and engagement with professional bodies facilitate the successful development and execution of immersive technology initiatives.

### Training and skill development

Training and skill development are important aspects of program implementation that begin with the familiarization of terminology associated with immersive technologies. Training should ideally include requirements development or the ability to identify opportunities for future iterations of software.

Implementation programs should also begin with onboarding to breed familiarization with program equipment and technology. It is suggested there be technical enablers, individuals who can guide new users through their experiences for users to grow accustomed to utilizing these technologies.

**Key takeaway:** Comprehensive training and onboarding are critical for building proficiency and ensuring the effective use of immersive technologies.

### Materials

Materials are critical to ensuring programs facilitate hands-on learning experiences. A variety of immersive devices, including headsets, hand controllers, and motion-tracking sensors, enable users to interact with virtual environments effectively. Equipment compatibility is essential to ensure all components work seamlessly together, enhancing the user experience.

Providing sanitization boxes for hygiene maintenance is paramount, especially in shared spaces. Wireless connectivity solutions contribute to flexibility and mobility, allowing users to engage with immersive content seamlessly without being tethered to a specific location. Ideally, there should be a 1:1 user-to-device ratio, such as headsets, or the program should include rotation terms and hygienic practices. Curating a comprehensive range of materials that consider compatibility, functionality, and hygiene ensures positive user experiences.

**Key takeaway:** Curating comprehensive materials that consider compatibility, functionality, and hygiene ensures positive user experiences.

### Leadership and education

A dedicated leadership and education support team with expertise in immersive technology devices, content creation, and delivery is essential. This team should assist students and program participants, ensuring the smooth integration of immersive technologies into the learning environment and promptly addressing technical issues.

Effective leadership and education require a clear vision and strategy, enabling leaders and instructors to seamlessly integrate technology into existing programs through curriculum design, assessment, and feedback mechanisms. Given the rapid pace of development in immersive technology, continuous learning and adaptation are vital. Establishing learning continuums is crucial to keeping pace with evolving trends and innovations, ensuring the program remains relevant and effective.

**Key takeaway:** Strong leadership and ongoing education support are critical for the smooth integration and continuous improvement of immersive technology advancements.

### Facilities

Facilities ideal for immersive technology use in medical teaching and training programs provide adequate space and resources to accommodate the hardware and software necessary for effective training. A dedicated room or space, away from distractions and noise, would provide an ideal environment for students to engage in immersive training. Many medical training and education programs have simulation centers ideal for immersive technology integration. This space should be equipped with comfortable chairs or standing tables that allow participants to remain engaged and focused on the training material for extended periods, but not usually more than an hour at a time.

The facility should also have access to high-speed internet and be equipped with the latest hardware and software. Equipment may consist of headsets, input devices, and other accessories necessary for effective immersive training. A robust network infrastructure should be in place to ensure a reliable connection between the immersive technology devices and the training material.

To achieve GHE subject-matter expert exchanges, the facility should also be equipped with robust virtual meeting capabilities. This should include video conferencing capabilities, virtual collaboration tools, and other technologies necessary for effective remote involvement. This would allow subject-matter experts from around the world to participate in the training and bring diverse perspectives to the table.

**Key takeaway:** Well-equipped facilities are essential for immersive technology training, providing an environment conducive to effective learning and international collaboration.

Implementing immersive technology in medical training programs requires a comprehensive framework encompassing doctrine, organization, training, materials, leadership and education, and facilities. Doctrine and policy provide clear vision and goals, ensuring all stakeholders understand the program’s objectives. Organizational structure delineates roles and responsibilities, fostering innovation and effective coordination. Comprehensive training and well-curated materials are essential for user proficiency and positive experiences. Leadership support and well-equipped facilities create an environment conducive to effective learning and global collaboration, ultimately enhancing global health outcomes and readiness. The following graph depicts the framework, illustrating the structured approach necessary for successful implementation.

The XR implementation and training framework depicted above provides a clear, tiered progression for integrating XR technologies into GHE initiatives across multiple domains. This framework details the evolution from a lack of capability to full and mature integration.

In the *doctrine/policy* domain, the framework begins with no recognition or support for XR, moving through stages that include gaining awareness, initial familiarization, and ultimately formal adoption as a teaching method with institutionalized programs and regular equipment updates.

For the *organization* domain, the trajectory starts with no centralized structure for XR, transitions to identifying leadership or a proponent for XR implementation, and concludes with active participation in international XR forums and collaborations.

In the *training* domain, it illustrates the journey from the absence of any program to the establishment of comprehensive training that is systematically maintained for all personnel involved in GHEs.

The *material* domain progresses from no available equipment or support for XR to complete access and logistical support for XR materials, ensuring that the necessary resources are in place.

*Leadership and education* development follows a similar pattern, starting from no trained staff, then moving through the development of professional programs, and concluding with full compliance and participation in training, ensuring leaders are equipped to guide XR implementation.

In terms of *personnel*, the framework describes the shift from having no designated personnel with XR expertise, through initial identification and training of individuals, to mandatory training and certifications required for personnel involved in XR initiatives.

Lastly, the *facilities* domain shows improvement from no dedicated spaces for XR activities, culminating in the establishment of regional facilities within hospitals that support sustained XR training programs.

Each of these domains follows a clear path from initial, often nonexistent, capabilities through various stages of development, ultimately reaching a comprehensive, mature state of XR integration.

The integration of XR technologies offers a transformative opportunity to enhance medical practice and training methodologies globally, supporting the principles of GHE. By bridging geographic gaps and democratizing access to specialized knowledge, the immersive technology framework facilitates the scalable delivery of education and health care services. Although the initial investment in XR equipment and software development may be significant, these technologies eliminate recurring expenses associated with physical training materials, travel, and accommodation for trainees and instructors. Immersive digital scenarios can be reused multiple times, enabling scalable and flexible online meeting sessions without incurring additional costs, while the initial investment in hardware and software is recuperated over time through their sustained utilization.^13^ Establishing a comprehensive framework tailored to the unique needs of diverse health care contexts allows for the systematic integration of technology to address pressing medical challenges and improve outcomes based on shared, identified requirements.

VR has been demonstrated to enhance surgical fellows’ self-confidence, thereby improving overall outcomes.^14,15^ The efficient execution of medical procedures is essential for minimizing both patient risks and financial expenditures. Extensive medical literature indicates that prolonged interventions are associated with higher rates of complications, extended hospital stays, and an increased need for subsequent treatments.^16–18^ These factors collectively strain health care resources and escalate costs. Consequently, optimizing medical interventions and training is imperative to address clinical urgency, enhance economic efficiency, and uphold ethical standards in medical practice.

As previously discussed, the convergence of XR and AI technologies offers promising opportunities for education and practice within the health care sector. The digital data required to develop educational and training environments do not necessarily need to originate from real patient information; instead, simulated data can be effectively utilized. In this context, generative AI plays a crucial role in producing synthetic data for simulation purposes, thereby unlocking new dimensions of opportunity.^19^ Specifically, synthetic data refer to artificially generated information that replicates the statistical characteristics of real-world data without containing personal health information or personal identifiable information. When integrated with XR immersion, these synthetic data enable health care professionals to engage in realistic, risk-free training scenarios, including rare or complex cases. Consequently, it enhances their skills and preparedness while avoiding concerns regarding patient privacy.

However, when MXR initiatives extend beyond education and training to incorporate real patient data or direct patient interventions, it is crucial to collaborate with regulatory bodies (e.g., the Food and Drug Administration (FDA) in the United States and Agencia Nacional de Vigilancia Sanitaria (ANVISA) in Brazil) to ensure compliance with established safety and efficacy standards. These initiatives must also adhere to stringent data protection regulations, including HIPAA in the United States, General Data Protection Regulation (GDPR) in Europe, and Lei Geral de Protecao de Dados (LGPD) in Brazil, to protect patient privacy and ensure the secure handling of sensitive information. The health care sector is highly regulated and characterized by its strict compliance with established guidelines and protocols, which aim to standardize treatment modalities and follow-up procedures. Nevertheless, different regions implement varied regulations, resulting in disparate outcomes. Integrating XR technology into health diplomacy aims to facilitate harmonious global collaboration, thereby advancing equitable and effective international health care outcomes.

Establishing a comprehensive framework for integrating XR into health care settings is essential for the routine adoption of these technologies within training programs, thereby significantly enhancing the preparedness of medical professionals for emergency situations. The immersive capabilities of MXR provide health care practitioners with boundless, realistic, hands-on experiences, effectively equipping them for high-pressure and time-sensitive environments.^1,2,9–12,14,15^ Although additional field studies are required, the utilization of XR technologies holds substantial promise for advancing training protocols, educational methodologies, and emergency response capabilities.

HealthTech BR, a pioneering startup in Latin America, effectively demonstrated the value of MXR by utilizing the region’s established simulation centers within medical schools. Confronted with the challenge of validating XR’s efficacy for medical training, the company strategically rented VR headsets to conduct trials of their multiuser immersive clinical simulation software. This practical approach enabled educators and students to engage with the enhanced learning opportunities provided by XR, culminating in the integration of HealthTech BR’s solutions into existing simulation laboratories alongside traditional mannequins. By focusing on medical schools rather than individual health care professionals, HealthTech BR is strategically addressing the high investment barriers that limit mainstream adoption of XR technologies in the region, laying the foundation for virtual-enabled training environments to participate in GHEs.

In contrast, countries, including the United States and various Eastern nations, have made substantial investments in advanced technology development, cultivating an environment where XR is fluidly integrated into training. Technological literacy and supportive innovation ecosystems in these regions enable early adoption and facilitate adoption and scalability of XR-based solutions.^20^ This comparison underscores the pivotal role that technological advancements play in shaping the global adoption landscape of immersive technologies as a foundation for medical education and GHE.

For health care training, advanced methodologies including clinical simulation, bridge the gap between theoretical knowledge and practical skills. It improves readiness for real-world scenarios by providing repetitive practice, immediate feedback, and scalable exposure to rare conditions. It bolsters confidence, refines procedural skills, and enhances patient safety.^21–23^ Integrating digital technologies into simulation training centers advances and complements medical education. Technology-enhanced simulation (T-ES), including VR, AR, and MR, offers scalable, accessible training that transcends traditional limitations.^24–26^ T-ES fosters global learning and knowledge sharing among medical practitioners, enhancing the efficiency of medical education. It allows health care students and professionals to gain insights into diverse clinical scenarios and systems. This global exchange of knowledge can promote standardized care and customized learning modules among different health care systems and professionals.

Digital simulations prepare professionals for facing diverse medical conditions and provide detailed feedback on performance, tracking progress over time. It democratizes medical education, making high-fidelity training accessible regardless of location. This innovation aims to enhance the skill set of medical professionals and improve global patient care. For developing countries, digital simulation can also address resource constraints by optimizing the use of materials and human resources. Through standardized training modules adhering to international best practices, digital simulation training ensures consistent medical care quality. Its scalability addresses the uneven distribution of medical education facilities, allowing professionals in remote areas to receive quality training. Continuous professional development is facilitated without the logistical and financial burdens of traditional training.

T-ES represents a transformative, cost-effective, equitable, and high-quality educational tool for medical training, ultimately contributing to improved patient outcomes. AR, VR, and MR are tools that can highly improve the fidelity of these digital simulations. Stakeholders interested in adhering to new methods for practical training in health care should observe meticulously the framework proposed for successful implementation.

## Conclusion

In conclusion, the authors and the professional work and associations to which they belong have a vision for employing spatial computing technologies to dramatically impact medical education and boost global health initiatives. By integrating VR, AR, and MR into training and operations, health care professionals are empowered to practice and collaborate beyond the traditional constraints imposed by geographic and logistic limitations. The next step for this technology to grow is to continue to make these systems accessible for more people through all areas of life and also to grow in making it accessible to enable distance communication. The goal of so many emerging technologies is to be able to collaborate and communicate from wherever you are in the world—in XR, it’s a way to virtually connect with friends, family, and other professionals. As a community, there is so much we can do to assist growth in the world by creating a better means of communication. To fully harness this potential, it is critical to adopt a framework that fosters standardization across training programs and aligns with global health objectives to assure technology development for optimized use, considering worldwide perspectives. This requires the adoption of new technologies to improve comfortability to proactively address integration challenges related to user experiences, system compatibility, and cultural diversity in technology use.

Moreover, the success of these technologies in advancing medical education and promoting international collaboration relies on overcoming several critical challenges. Future research should focus on enhancing haptic feedback to improve the tactile experience essential for medical training. Addressing other issues such as cybersickness, user acceptance of new technologies, and the effective evaluation of those experiences. Also, the effective integration of immersive technologies as didactic tools necessitates substantive engagement from academic experts and proficient practitioners. The concerted involvement of seasoned health care professionals is pivotal in steering the evolution of such technologies toward becoming cohesive adjuncts to curricular frameworks. Network and communication issues, along with traffic management, also present substantial obstacles that can affect the efficiency and scalability of immersive technology applications. Additionally, ensuring robust cybersecurity measures and developing user-friendly hardware controls are essential for maintaining the security and accessibility of spatial computing systems.

Successful implementation will depend on continuous innovation, thoughtful adaptation based on user feedback, and proactive engagement with international partners. Addressing these challenges is key to unlocking the transformative potential of immersive and related technologies, thereby significantly advancing health care outcomes worldwide. By expanding the accessibility and effectiveness of medical training, these technologies promise to make a profound impact on GHE and readiness, setting a course for sustained collaboration and continual improvement among international health care professionals.

## Abbreviations Used

AI: artificial intelligence
AMXRA: American Medical Extended Reality Association
ANVISA: Agência Nacional de Vigilância Sanitária
AR: augmented reality
CCPA: California consumer privacy act
CPR: cardiopulmonary resuscitation
DHA: defense health agency
DoD: department of defense
DoDI: department of defense instruction
DOTMLPF-P: doctrine, organization, training, Material, leadership, personnel, facilities, and policy
FDA: food and drug administration
GDPR: general data protection regulation
GHE: global health engagement
HIPAA: health insurance portability and accountability act
IHVRA: international healthcare virtual reality association
LGPD: Lei Geral de Proteção de Dados
MR: mixed reality
MXR: medical extended reality
PN: partner nation
SBIR: small business innovation research
T-ES: technology-enhanced simulation
VR: virtual reality
XR: extended reality
